# Three-dimensional Analysis of Facial Asymmetry in Unilateral Lambdoid Craniosynostosis

**DOI:** 10.1177/10556656231176876

**Published:** 2023-05-17

**Authors:** Lucas M. Harrison, Eliza J. Ferrari, Denzil P. Mathew, Christopher A. Derderian, Rami R. Hallac

**Affiliations:** 1 Department of Plastic Surgery, University of Texas Southwestern Medical Center, Dallas, TX, USA; 2 Analytical Imaging and Modeling Center, Children’s Medical Center, Dallas, TX, USA

**Keywords:** craniosynostosis, craniofacial morphology, facial morphology

## Abstract

**Objective:**

Unilateral lambdoid synostosis (ULS) is characterized by occipital flattening, mastoid bulging, and contralateral parietal bossing. Anterior craniofacial features are less well-defined. This study utilizes volumetric, craniometric, and composite heat maps of three-dimensional (3D) rendered CT scans to analyze anterior craniofacial asymmetry in ULS and compared to controls.

**Design:**

A retrospective review of three-dimensional CT scans.

**Setting:**

Tertiary care pediatric institution.

**Patients, Participants:**

30 ULS and 30 control patients.

**Main Outcome Measure(s):**

Volumetric and craniometric analysis of the anterior fossa, orbits, zygomas, maxilla, and mandible was performed.

**Results:**

The anterior fossa volume was greater bilaterally (0.047, 0.038), and the fossa angle was more anterior contralaterally (<0.001) and more anterior bilaterally than controls (0.038, 0.033). The orbits had greater height and lesser depth bilaterally compared to controls (0.006, 0.009; < 0.001, < 0.001). Zygoma length was significantly greater on the contralateral side than controls (0.048; < 0.001). Nasal contralateral deviation of 3.57  ±  1.97°. The maxillary length was longer on the contralateral side (0.045). The mandibular angle was more anterior on the ipsilateral side and posterior on the contralateral side (<0.001) compared to controls (0.042, < 0.001). Chin had a contralateral deviation of 1.04  ±  3.74°.

**Conclusions:**

ULS has significant asymmetry in the anterior craniofacial skeleton. There is a bilateral expansion of the anterior cranial fossa with greater frontal bossing on the contralateral side. Increased orbital height and decreased depth. Contralateral zygomatic and mandibular body lengthening with posterior mandibular deviation. These features may provide more effective diagnosis and potential clinical management strategies.

## Introduction

Unilateral lambdoid synostosis is the least common form of single suture craniosynostosis, accounting for only 1 to 4% of all cases.^1^ Commonly agreed upon features of ULS include ipsilateral occipital flattening, occipitomastoid bulging, and inferior canting of the skull base on the ipsilateral side with contralateral posterior parietal bossing.^1–13^ Consistent clinical observation of anterior craniofacial asymmetry has often been described. However, there exists a dearth of comprehensive quantitative analysis.

Most of the morphometric studies evaluating unilateral lambdoid synostosis have primarily focused on alterations in the middle and posterior cranial vault; a few small studies have begun to describe some of the anterior craniofacial features. Previously, the laterality of frontal bossing had been used in the differentiation of synostosis and deformational plagiocephaly.^2–5^ However, recent studies have suggested the laterality of frontal bossing to be a less predictable feature.^6–8^ The maxillary depth and zygoma length have also been shown to be increased in the contralateral midface.^8,9^ In the lower face, the contralateral articular fossa of the mandibular condyle is displaced anteriorly, leading to a facial twist.^8,10^

Features of facial asymmetry appear to persist following surgery; therefore, it is worthwhile to perform a comprehensive analysis to understand better the complex anterior deformational changes in this rare condition.^7^ We hypothesized that our analysis would demonstrate anterior craniofacial asymmetry to support our clinical findings in patients with ULS. This study provides volumetric, craniometric, and composite heat map analysis of anterior craniofacial asymmetry in unilateral lambdoid synostosis patients and comparison to controls using preoperative three-dimensional rendered computed tomography (CT) scans.

## Methods

Retrospective CT scan analysis of 30 patients diagnosed with ULS following institutional review board approval by the University of Texas Southwestern Medical Center. All CT images were obtained pre-operatively, and no patient with a synostosis-related syndrome, positional plagiocephaly, or any other craniofacial malformation was included. An additional 30 age- and gender-matched control subjects’ images without pathological findings were analyzed for comparison. The average age of the ULS group was 1.84  ±  2.25 years and the control group were 1.85  ±  2.24 years. In both groups 16 were male and 14 were female. Right sided laterality was present in 17 patients and left sided in 13. Metopic ridge was present in 13 patients. CT images were converted to three-dimensional skeletal models using Mimics 22.0 (Materialise, Belgium) and then analyzed using 3-Matic 15.0 (Materialise, Belgium).

### Volumetric Analysis

**Figure 1.**
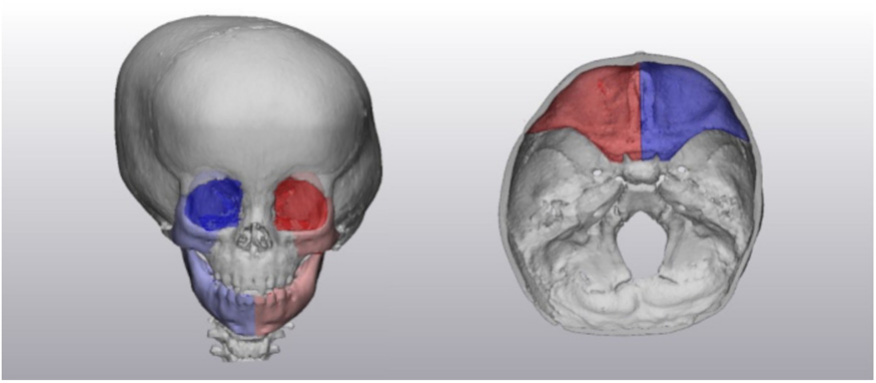
Volumetric segmentation of the orbit, zygoma, mandible, and anterior cranial fossa.

Volumetric data was collected from 3-Matic 15.0 (Materialise, Belgium). The anterior fossa volume was separated by tracing along the edge of the lesser wings of the sphenoid bone to the edge of the cranial vault. The anterior fossa was further split into left, and right-sided volumes separated by a plane through the anterior nasal spine and sella turcica (Figure 1). The orbital volumes were determined by segmentation of the orbital aperture (Figure 1). Zygoma volume was segmented along the frontozygomatic suture, zygomaticotemporal suture, and the zygomaticomaxillary suture (Figure 1). Mandibular volume was separated into left and right along the symphysis (Figure 1).

### Craniometric Analysis

**Figure 2.**
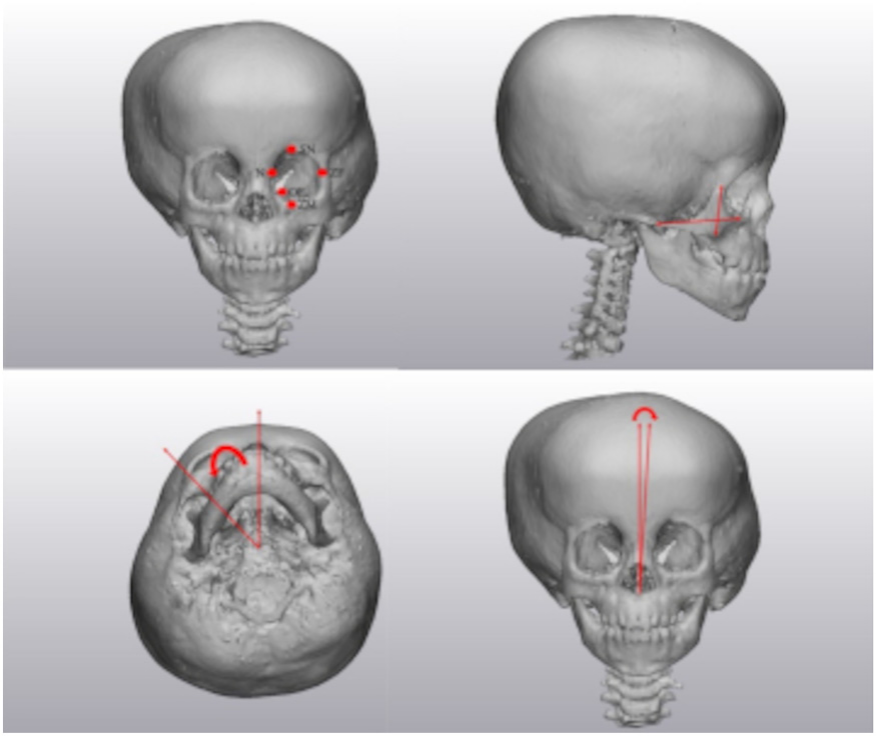
Orbital landmarks (top left), zygomatic measures (top right), zygomatic angle (bottom left), and nasal deviation (bottom right).

**Figure 3.**
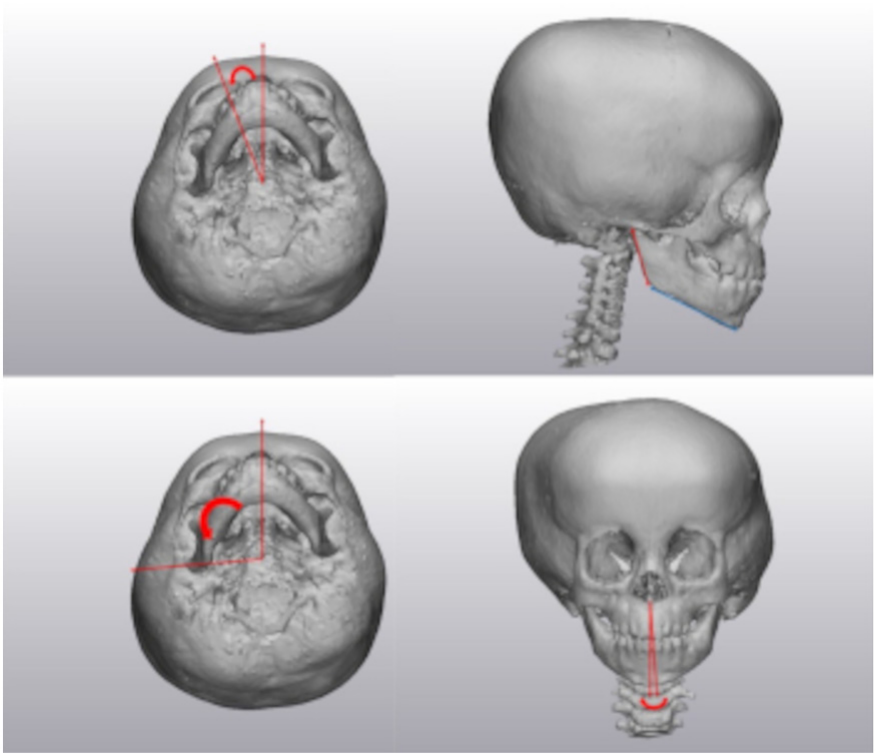
Maxillary angle (top left), mandibular lengths (top right), mandibular angle (bottom left), and chin deviation (bottom right).

**Figure 4.**
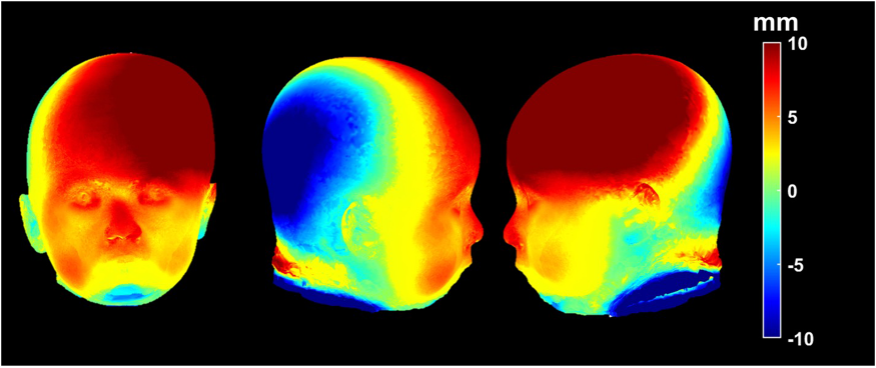
Heat maps demonstrating areas of difference between composites of right sided unilateral lambdoid and control patients. Warmer colored areas representing projection and cooler colored areas representing retrusion.

The three-dimensional rendered skeletal models were analyzed by measuring various craniometric distances and angles. The anterior fossa angle was measured between the mid-sagittal plane and the lesser wing of the sphenoid (Figure 2). Orbital measurements included height (zygomaticomaxillary (ZM)-supraorbital notch (SN)), width (zygomaticofrontal (ZF)—nasion (N)), depth (ZM—optic foramen (OF)), horizontal angle (ZF-OF-N), and vertical angle (ZM-OF-SN) (Figure 3). Zygomatic measurements included length (ZM-zygomaticotemporal suture (ZT)), height (ZF-lowest point of zygoma), and angle (midsagittal plane-sella-ZM) (Figure 3). The nasal deviation was measured from nasion to anterior nasal spine (ANS) to midsagittal plane (Figure 3). Maxilla was measured by angle (midsagittal plane-sella-pterygoid hamulus) (Figure 4). Mandibular measurements included ramus length (notch-angle), body length (angle-parasymphysis), and angle (midsagittal plane-sella-articular fossa (Figure 4). Chin deviation was measured from midsagittal plan to ANS to symphysis (Figure 4).

### Composite Heat Map Analysis

Composite (average) heads were calculated using previously described mathematical models.^14,15^ The magnitudes of three-dimensional vectors from a center point of the head (between the two tragi) to each point on the three-dimensional surface were calculated for the lambdoid group and were compared to the control group. A heat map was generated to represent the morphological difference between the lambdoid and control subjects.

### Statistical Analysis

All craniometric distances and angles were collected in triplicate. The average value and standard deviation were then calculated. Two different authors collected the measurements independently and then averaged their values. Measurements were obtained in an unblinded fashion but were corroborated with blinded analysis. Statistical analysis between groups was performed using two-sample unpaired student t-tests with statistical significance considered a value of *p* < 0.05.

## Results

The anterior intracranial volume distribution is shown in Table 1. Substantial volume expansion of the bilateral anterior fossae was observed in ULS compared to the control group. There was no significant difference in the volume distribution between the anterior fossa of the ipsilateral and contralateral sides. The ipsilateral fossa angle was significantly greater, indicating a more posterior deviation than the contralateral side and control. The contralateral side was significantly less, indicating a more anterior deviation than control.

**Table 1.** Upper, Middle, and Lower Facial Third Measurements (Mean  ±  STD).

Upper Facial Third	Ipsilateral	Contralateral	Control	Ipsilateral vs Contralateral	Ipsilateral vs Control	Contralateral vs Control
Anterior Fossa						
Fossa Volume	99.62 ± 31.13 cm^3^	101.87 ± 35.83 cm^3^	86.90 ± 30.25 cm^3^	0.800	**0**.047*	**0**.038*
Fossa Angle	77.76 ± 3.82°	73.19 ± 2.86°	75.22 ± 3.48°	**<0**.001*	**0**.033*	**0**.038*
Middle Facial Third	Ipsilateral	Contralateral	Control	Ipsilateral vs Contralateral	Ipsilateral vs Control	Contralateral vs Control
Orbit						
Orbital Volume	14.66 ± 2.96 cm^3^	15.09 ± 3.18 cm^3^	13.96 ± 2.95 cm^3^	0.694	0.444	0.243
Orbital Height	30.05 ± 1.91 mm	30.30 ± 2.30 mm	28.36 ± 2.18 mm	0.929	**0**.006*	**0**.009*
Orbital Width	30.05 ± 1.91 mm	30.52 ± 1.99 mm	30.42 ± 2.44 mm	0.501	0.566	0.884
Orbital Depth	32.34 ± 2.72 mm	32.72 ± 2.42 mm	35.70 ± 2.66 mm	0.929	**<0**.001*	**<0**.001*
Horizontal Orbital Angle Vertical Orbital Angle	55.74 ± 4.31° 53.21 ± 2.90°	57.46 ± 3.85° 53.43 ± 3.10°	54.67 ± 2.25° 45.52 ± 2.30°	0.241 0.835	0.367 <0.001*	0.014* <0.001*
Zygoma						
Zygomatic Volume	1.85 ± 0.63 cm^3^	1.93 ± 0.70 cm^3^	2.09 ± 0.64 cm^3^	0.731	0.219	0.446
Zygomatic Length	47.16 ± 5.31 mm	51.01 ± 5.24 mm	42.78 ± 5.17 mm	**0**.048*	**0**.012*	**<0**.001*
Zygomatic Height	29.01 ± 4.11 mm	28.55 ± 3.90 mm	28.49 ± 4.46 mm	0.749	0.696	0.964
Zygomatic Angle	45.78 ± 2.69°	45.17 ± 2.74°	44.42 ± 1.65°	0.531	0.079	0.327
	Deviation Ipsilateral Side			Deviation Contralateral Side		
Nasal Deviation	−			3.57 ± 1.97°		
Lower Facial Third	Ipsilateral	Contralateral	Control	Ipsilateral vs Contralateral	Ipsilateral vs Control	Contralateral vs Control
Maxilla						
Maxillary Angle	32.36 ± 4.84°	33.21 ± 4.14°	30.19 ± 4.03°	0.608	0.530	0.201
Mandible						
Mandibular Volume	5.38 ± 1.71 cm^3^	5.76 ± 1.77 cm^3^	6.01 ± 1.38 cm^3^	0.802	0.613	0.842
Ramus Length	28.57 ± 3.96 mm	28.87 ± 4.75 mm	30.05 ± 4.26 mm	0.881	0.462	0.586
Body Length	40.28 ± 3.41 mm	44.70 ± 4.46 mm	42.97 ± 3.19 mm	**0**.031*	0.113	0.369
Mandibular Angle	94.83 ± 4.83°	102.51 ± 3.72°	95.85 ± 6.02°	**<0**.001*	0.536	**<0**.001*
	Deviation Ipsilateral Side			Deviation Contralateral Side		
Chin Deviation	−			1.04 ± 3.74°		

(*) Statistical significance.

The results of the orbital measurements are shown in Table 1. Orbital volume and orbital width were not significantly different between groups. The orbital height and vertical orbital angle were significantly increased on both sides in ULS patients compared to controls. There was no significant difference in horizontal orbital angle, but the vertical orbital angle was greater on both sides than controls. Orbital depth on both the ipsilateral and contralateral sides was significantly less than controls. Zygomatic volume and angle were not significantly different between sides or to control. The zygomatic length was significantly longer on both sides in ULS than controls, and the contralateral side was significantly longer than the ipsilateral. There is nasal deviation towards the contralateral side.

The maxillary angles were not significantly different between sides and compared to control. Mandibular volume was not significantly different shown in Table 1. Mandibular ramus length was not significantly different, but body length was significantly longer on the contralateral side than on the ipsilateral. The mandibular angle was significantly more significant on the contralateral side, indicating more anterior projection than on the ipsilateral side or control.

Heat maps created from overlaid right sided ULS and control patient group composites are shown in Figure 4. The areas of greatest difference are identified with color-coding, with warmer colors demonstrating areas where ULS patients show more projection and cooler colors demonstrating areas where they show more retrusion.

## Discussion

Premature closure of the lambdoid suture profoundly affects skull growth, resulting in significant asymmetry of the cranial base, calvarium, and facial skeleton. Although many of the characteristic deformities in ULS are well documented in the literature, previous studies have primarily focused on calvarium and cranial base morphological changes. The current study provides a comprehensive and quantitative analysis of the anterior craniofacial asymmetry in ULS patients, employing a combination of volumetric, craniometric, and composite heat map analysis using preoperative three-dimensional rendered CT scans. This approach allows for a more detailed and accurate assessment of the anterior craniofacial features in ULS patients than previously reported. The relatively large cohort of 30 patients enables us to draw more robust conclusions regarding anterior craniofacial asymmetry in ULS patients. These novel findings enhance our understanding of this rare condition's complex anterior deformational changes, ultimately informing more effective diagnosis and potential clinical management strategies.

Reports of anterior cranial morphology in ULS have been inconsistent in previous studies and have described ipsilateral and contralateral frontal bossing cases.^7^ Our volumetric data demonstrated symmetric volumetric expansion of both anterior cranial fossae. Bilateral frontal bossing is appreciable in the 3D heat map images in which the bilateral forehead has increased projection compared to controls, indicated by the warm solid color of the region. These images also show a slighter greater vertical and lateral projection of the forehead on the contralateral side than on the ipsilateral side. This finding may account for previous reports that frontal bossing only occurs on the contralateral side, described in studies that did not compare subjects with ULS to normal controls.

While orbital dysmorphology is a documented feature in craniosynostosis of other major sutures, particularly metopic and coronal synostosis, this has not previously been described in lambdoid synostosis.^13,14^ We found that orbital height was increased, and orbital depth was reduced bilaterally in patients with ULS compared to controls. These changes also corresponded to an increased vertical orbital angle bilaterally compared to controls. The taller orbit in subjects with ULS may be related to the expansion of the bilateral anterior cranial fossae. Increased anterior intracranial volume may favor anterior and vertical compensatory growth of the bony orbits, resulting in altered dimensions of the orbit without an absolute change in total orbital volume. A similar but more severe pattern of increased anterior and vertical compensatory growth of the anterior cranial vault associated with increased vertical dimensions of the orbits is observed in Mercedes-Benz pattern craniosynostosis.^17^ Decreased orbital depth may be due to anterior displacement of the middle fossae from restricted posterior fossa growth, but this was not evaluated in the current study.

When examining the midfacial structures, the zygoma length was significantly longer on the contralateral side compared to the ipsilateral side. This indicates midface protrusion on the contralateral side of the face in ULS patients. This had previously been theorized by Smartt et al., who speculated that anterior displacement of the mandible on the ipsilateral side might coincide with anterior displacement of the midface and lengthening of the zygoma on the contralateral side.^6,8^ This was later supported by Omar et al., who reported ipsilateral shortening and contralateral lengthening of the zygoma in their cohort of ULS patients.^16^ In the patients with ULS there was nasal deviation towards the contralateral side of suture closure at an average of 3.5 degrees and visibly noticeable on composite image, further supporting the findings of a facial twist appearance.

The results of the lower facial analysis are consistent with the findings of previous studies and demonstrate that mandibular angles at the articular fossa were all increased on the contralateral side, which appears to reflect a posterior shift of the cranial base on the contralateral side.^5,8^ Growth away from the occipitomastoid bulge on the ipsilateral side may be responsible for the anterior displacement on the ipsilateral side. The mandibular body was significantly longer on the contralateral side, indicating compensatory changes due to posterior displacement of the contralateral articular fossa. This contradicts previous findings by Smartt et al., who found the body length similar between sides in their cohort of 9 patients with ULS.^8^ The chin was also deviated toward the contralateral side by 1 degree. Ploplys et al. noted the presence of “facial twist” in ULS patients, manifesting as 1 degree of chin deviation towards the contralateral side.^5^

Notably, 43% (13 out of 30) of our patients with ULS exhibited visible metopic ridging on skeletal CT images. Benign metopic ridge is thought to be present in 10-25% of infants as a variant of normal, which raises the question if the increased incidence in our study population is more than coincidental.^18^ The potential implications of this finding warrant further investigation to understand the relationship with ULS and its impact on the anterior cranial fossa changes. One potential explanation is that the metopic ridge could be related to the bilateral frontal bossing we observed in these patients. Frontal enlargement could theoretically disrupt bone remodeling processes in the metopic suture, resulting in a more prominent ridge. Interestingly, Fisher et al. reported an increased incidence (39%) of metopic suture anomalies in patients with deformational plagiocephaly.^19^ The authors hypothesized that mechanical forces on the posterior skull might be responsible for metopic ridging in these patients. A similar mechanism may be relevant in patients with ULS. Although our current study did not specifically analyze the anterior cranial fossa changes associated with a metopic ridge, we acknowledge that these changes may confound the interpretation of our findings in ULS patients. Future studies should consider performing a subgroup analysis comparing ULS patients with and without metopic ridging to better understand the potential impact of this association on the craniofacial features observed in our cohort.

Traditionally, surgical correction for ULS has focused on reshaping the posterior cranium. Depending on the patient's age, this may involve endoscopic strip craniectomy or open posterior cranial vault remodeling.^1^ Although surgery improves overall head shape in ULS, anterior skull deformities such as frontal bossing, orbital, zygomatic, and mandibular changes may not be effectively treated with the current surgical techniques. Additionally, these deformities of the anterior craniofacial skeleton may persist after surgery. Smartt et al. described a postoperative “hemifacial deficiency” in ULS patients.^6,8^ While not as dramatic as the posterior cranial asymmetry seen in ULS, these residual facial deformities could be distressing to patients.

Our findings highlight the importance of considering the complex anterior craniofacial deformations in patients with ULS when planning surgical management. Although our study does not explicitly propose a surgical technique or staged procedures, our results contribute to the ongoing discussion about the most appropriate surgical management for ULS patients. By incorporating a comprehensive understanding of anterior craniofacial asymmetry into the decision-making process, clinicians can develop more targeted and individualized treatment plans that address each patient's specific needs. Future research should focus on evaluating the outcomes of various surgical approaches in the context of these findings.

## Conclusions

Unilateral lambdoid synostosis results in a significant asymmetry in the anterior craniofacial skeleton. There is compensatory contralateral superior and midface protrusion found with frontal bossing and increased zygomatic protrusion. Orbital height expansion with decreased depth. There exists midline deviation toward the contralateral side in both the nose and the chin. In the lower third the ipsilateral side has increased protrusion in the mandible and in the body length. An increased incidence of the benign metopic ridge was also observed in our cohort. These findings in the anterior cranium, orbits, midface, and mandible should be considered for diagnosis and potential clinical management strategies.
